# 125 years of head and neck radiotherapy: could organ-sparing radiotherapy of larynx cancer have prevented World War I?

**DOI:** 10.1007/s00066-022-01902-9

**Published:** 2022-02-11

**Authors:** Thomas B. Brunner, Herbert Wördehoff, Ahmed Gawish, Uwe Busch

**Affiliations:** 1 Department of Radiation Oncology, Otto-von-Guericke University of Magdeburg, Leipziger Str. 44, 39120 Magdeburg, Germany; 2 City of Remscheid, German Roentgen-Museum, Remscheid, Germany

**Keywords:** Diagnosis, Celebrity, History, Primary therapy, Glottic tumors

## Abstract

**Purpose:**

We aim to recapitulate the rapid development of head and neck radiotherapy in the context of otorhinolaryngology (ORL) medicine starting 125 years ago. This is put into context with the unsuccessful treatment of the laryngeal cancer (LC) of the German emperor Frederick III and its historical consequences.

**Methods:**

The three-step process consisted in the analysis of (1) historical sources of the development of ORL radiotherapy from the discovery of x‑rays and radioactivity until World War I, (2) course and treatment of Frederick’s III LC, (3) political context with a special focus on the escalation towards World War I. Pertinent historical illustrations of technical developments of radiotherapy were summarized in a video.

**Results:**

ORL radiotherapy initiated on 03 February 1896, only 65 days after the discovery of X‑rays. By 1914, organ-sparing LC radiotherapy was established with a predominance of curietherapy over roentgentherapy. Correct diagnosis of Frederick III’s primarily radiocurable cT1a glottic LC was delayed by one year, which resulted in advancement to a fatal pT4 pN1 Mx tumour stage. Historically, his successor, William II, was assumed to have contributed to the causes of World War I.

**Conclusion:**

ORL radiotherapy came only eight years late to treat Frederick III who might have impeded World War I. This illustrates the potential impact of modern curative radiotherapy on the future course of public life beyond the personal fate of the patient himself.

**Video online:**

The online version of this article contains one video. The article and the video are online available (10.1007/s00066-022-01902-9). The video can be found in the article back matter as “Electronic Supplementary Material”.

## Introduction

Today organ preservation therapy of early stage larynx carcinoma is standard [1]. Yet, we tend to underestimate the fact that larynx cancer is the prime example of organ-sparing treatment with radiotherapy which was developed with tremendous speed after the discovery of x‑rays and of the nuclear physics of radium. This is even more intriguing as larynx cancer was regarded to be generally fatal throughout the 19th century, and only gradually during the 20th century did long-term cure rates rise from 4% to 44% to the current 64% (https://www.nuffieldtrust.org.uk/resource/cancer-survival-rates#background).

Technical progress of the Belle Epoque (Wilhelminismus) in the late 19th century and early 20th century until the start of World War I allowed for important steps forward in medicine [2, 3]. At the same time this era was responsible for new military possibilities which became manifest in the years from 1914 onwards. Almost immediately after the discovery of x‑rays and radioactivity, diagnostic and therapeutic exploitation of these physical milestones started. One of the first therapeutic applications in oncology was 125 years ago: the first documented radiotherapy of larynx cancer, a common type of cancer due to the high prevalence of smoking at that time [4].

## Materials and methods

A dual retrieval approach of historical sources between 1800 and 1925 was chosen on one the hand on the development of milestones in head and neck cancer treatment and radiotherapy. On the other hand the search comprised the biography of emperor Frederick III and the political circumstances of his life and thereafter until the declaration of war in 1914. The search not only comprised text sources but also pictorial sources in conjunction with the German Röntgen Museum in Lennep, Remscheid. Further developments of radiotherapy after World War I were not taken into account and excluded from the analysis.

## Results

### Discovery of x-rays and of radioactivity

The foundations for radiotherapy were laid within three years between 1895 and 1898. On 30 November 1895, Wilhelm Conrad Röntgen discovered x‑rays in Würzburg, Germany, in his laboratory at the Julius-Maximilians-University [5]. He was working with a gas ion tube, a partially evacuated glass tube that contained an induction coil. He had covered the tube with black paper and created conditions of complete darkness in the room. He observed that an electrical discharge from the tube produced an illumination of a paper screen which was randomly close to the gas ion tube and covered with the fluorescent material barium platinocyanide. Realising that neither the black paper nor other materials obstructed the effect, but when he put his hand between the tube and the screen he saw the bones of his hand projected upon the screen. On 28 December 1895 he submitted his manuscript to the secretary of the Physikalische Medizinische Gesellschaft of Würzburg for publication in the annuals of the society.

Less than half a year later, the physics professor Antoine Henri Becquerel (1852–1908) discovered radioactivity on 1 March 1896. The intention was to test whether x‑rays were produced by fluorescence. Earlier, he had put fluorescent mineral crusts on photographic plates which were wrapped in a light-impervious black paper to expose them to sunlight, and to see whether there are images on the plates. Indeed, he observed the expected images. However, in February 1896 dim sunlight conditions caused him to put the crusts on top of the photographic plates into a drawer without prior exposition to light. Oddly, for unknown reasons, on 1 March he took them out of the drawer to immediately develop the photographic plates. Becquerel was surprised to see images of the crust shapes on the developed plates. Within 10 days he published his observation in a paper in the *Comptes Rendus* [6] and within 3 months after his discovery he proved that uranium was the source of the radiation. Becquerel cooperated with Pierre and Marie Curie who focused on pitchblende in 1898 to isolate in July of that year radium as a new radioactive element [7].

Before focusing on larynx cancer, we would like to give a brief overview of the early development of radiation oncology in general. X‑ray treatment of cancer started with the treatment of head and neck tumour as described below. The first verifiable report at a medical meeting was given by Sjögren and Steenbeck at a meeting of the Swedish Society of Medicine on 19 December 1899 [8]. One patient was treated for a basal cell carcinoma of the nose and the other for squamous cell carcinoma of the cheek with histological proof for both tumours alongside with photographs at follow-up. A pioneering X‑ray textbook from Germany from 1903 described cancer and sarcoma radiotherapy and differentiated superficial lesions from deep lesions requiring soft versus hard x‑rays mostly focusing on palliative treatment indications [8]. In 1913, x‑ray therapy became significantly more robust by the introduction of Coolidge tubes with a hot cathode facilitating the era of deep x‑ray therapy [9]. Parallel with the development of x‑ray radiotherapy, radium treatment or curietherapy evolved. In 1903, two patients with basal cell carcinoma of the face were reported to have been treated successfully by Goldberg and London from St Petersburg [10]. In the same year, Strebel pioneered an interstitial technique in Munich with radium sources in an afterloading technique. The first applicators for curietherapy of cancer of the cervix date from 1905 in Paris. Another form of curietherapy was the use of radon seeds which were used in many tumour sites starting in 1908.

### Early reports of radiotherapy of laryngeal cancer—the first two decades

Only three months after the discovery of x‑rays by Roentgen, the first treatment of head and neck cancer was performed by Dr Voigt in Hamburg before 3 February 1896 (Table 1). He reported to the Society of Physicians of Hamburg to have successfully treated a patient with inoperable pharyngeal carcinoma with palliative intent for pain relief. However, Voigt did not publish this in a medical journal [11]. Nevertheless, Leopold Freund (1868–1943) described the appearance of a severe skin reaction in this patient subsequent to therapy that appeared around the time when the patient died [12]. At that time, the maximum energy available for the production of x‑rays ranged between 50–100 kV with an electric current far below 1 mA, thus leading to extremely long exposure times [13].

**Table 1:** Milestones of x‑ray treatment for head and neck cancer during the early years. Modified from Lederman [4]

Date of first treatment	Year of publication, author country	Comment
Before 3 February 1896	1896 Voigt, Germany [11]	Nasopharynx cancer, Relief of pain (case report)
October–December 1901	1904 Dobson, England [14]	Larynx cancer, ca. 2 years survival (case report)
October 1901	1902 Leland, USA [15]	Meeting report on larynx cancer
16 January 1902	1902 Dawson Turner, Scotland [16]	Neck glandular tuberculosis treated with x‑rays, no tumour response
3 March 1902	1903 Lowe, England [17]	Head & neck tumours, several cases of clinical complete responses
March 1902	1902 Delavan, USA [18]	Larynx cancer, several cases
Before May 1902	1902 Fletcher, USA [19]	Meeting report on larynx cancer
Before May 1902	1902 Payson, USA [20]	Meeting report on larynx cancer
Mid-June 1902	1902 Scheppergrell, USA [21]	Left vocal cord cancer, clinical complete response for 2 years
4 July 1903	1904 Massier, France [22]	Larynx cancer, case report
1 December 1903	1904 Béclère and Viollet, France [23]	Larynx cancer and maxillary sarcoma, clinical complete responses in both cases
3 January 1905	1906 Mader, Germany [24]	Intracavitary x‑rays
July 1906	1906 Knipers, The Netherlands [25]	Larynx cancer, case report

It is not surprising that the larynx was one of the first organs to be treated with radiotherapy and the first physician to perform radiotherapy for larynx cancer is thought to be Dobson from London in 1901 [14]. After initial dermatologic observations of the effects of x‑rays due to the absence of obstacles between x‑rays and the skin and the possibility to administer high doses to the skin, the larynx was accessible to diagnostic tools of the Victorian times allowing visualisation of pathologies, access for biopsies and pathologic work up of specimens and also to therapeutic approaches. Therefore, the application of x‑rays to diseases of the larynx were the logical next step. Nevertheless, this prime example of early radiation oncology also reflects the challenges that had to be overcome to develop a robust, effective and safe therapeutic modality. Though diseases of the larynx manifest themselves at an early stage most commonly with hoarseness, and cancers of the larynx tend not to spread early to lymph nodes, the organ is situated in a vulnerable place: All therapeutic approaches need to respect the function of the upper airways and to maintain the oral intake of nutrition in the pharynx and the cervical oesophagus. The protection of the anatomical and physiological specificities of the larynx was even more an enormous challenge in the early days of radiotherapy as there was no radiobiologic knowledge, dosimetry and technical devices for the administration of radiotherapy to the tumour. Such understanding and technical progress was only made in the 1920s by Claudius Regaud (1870–1940), Henri Coutard (1876–1950) and Albert Hautant (1887–1947) at the Institute Radium, Paris, which has to be regarded as the birthplace of organ preservation radiotherapy of the larynx [26].

**Fig. 1:**
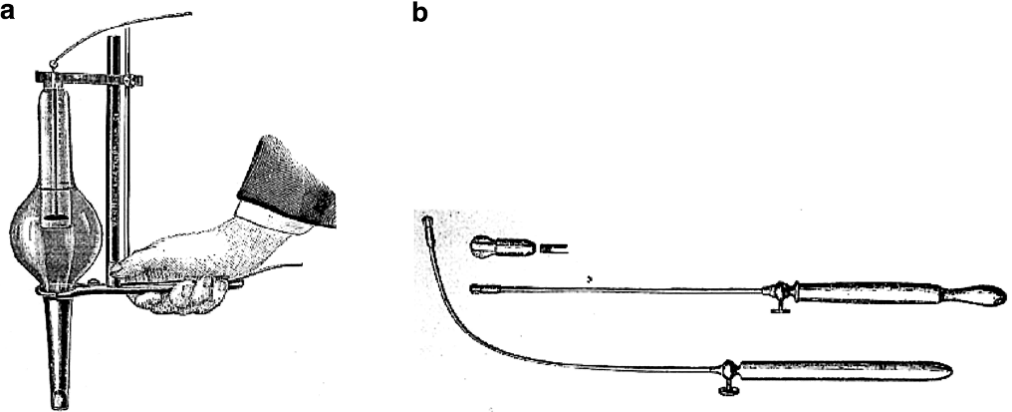
**a** Drawing of an applicator for intracavitary treatment of larynx cancer with an x‑ray tube described: “Handle, shield, short-circuiting switch, and tube.” (From: Pusey and Caldwell [27]) **b** Brachytherapy applicator with radium as used by Delsaux [31] for cancer of the larynx and other head and neck tumours. (From: [32])

The milestones between the discovery of the x‑rays and the refined treatment as described by Regaud and colleagues in 1922 are described in short here: One major step for x‑ray therapy was the development of specific intracavitary treatment tubes for larynx cancer by 1904 (Fig. 1a; [27]). The problems were that the accurate application was difficult and that long treatment times made it hard for the patients to tolerate radiotherapeutic sessions. The next major progress in radiotherapy of larynx cancer was the introduction of brachytherapy (Table 2). After the discovery of radioactivity by Becquerel in 1895, Marie and Pierre Curie were successful to isolate radium in 1898 which came from pitchblende (uraninite) from the Ore Mountains in Jáchymov/Joachimsthal. One of the early days problems of brachytherapy was access to radium salt which was rare and difficult to obtain. Therefore, other isotopes such as mesothorium and radon gas were tried to circumvent this shortcoming as they were cheaper and more frequent. The first reported case of radioactive treatment of head and neck cancer is from 1904 by Sir Mackenzie Davidson (1856–1919) in England. A dedicated radium carrier to treat larynx cancer was designed by Delsaux in 1903 (Fig. 1b; [28]). The fact that progress in the development of proper x‑ray devices was slow, promoted the development of brachytherapeutic application methods. This comprised intracavitary techniques with radium tubes, e.g. by Delsaux and refined models, such as the Dominici tube [29]. The latter included a 0.1 mm platinum filtration to shield harmful alpha and beta radiation and to allow only pure gamma radiation to be emitted. The source was an anhydrous radium sulphate in a small sealed platinum cell. In 1909, the Englishman Neville Samuel Finzi (1881–1968) was the pioneer of external use of radium [30]: with two 50 mg radon tubes attached to each side of the larynx he achieved good results in patients with larynx cancer.

**Table 2:** Milestones of curietherapy during the first two decades. Modified from Lederman [4]

Date of first treatment technique	Year of publication, author, country	Comment
July 1903 Suggested radioactive catheter	1904 Mackenzie, England [33]	Cancer of face and pharynx (several cases)
Before August 1903	1903 Delsaux, Belgium [31]	Tuberculosis of larynx
Before May 1904	1904 Delsaux, Belgium [34]	Carcinoma larynx (2 cases)
Before August 1904 radium	1904 Beck, USA [35]	Benign and malign diseases, unsatisfactory responses (11 cases)
1907	1913 Abbe, USA [36]	Papilloma of the larynx
1908 Description of radium applicator	1908 Nicolai, Italy [37]	Tuberculosis of the larynx (cases)
1909 Reported radium catheter	1909 Wickham & Degrais, France [38]	Parotid tumour, 9 cm diameter (complete response); neck nodes (partial response) (2 ENT cases)
1909 Reported radium tubes applied externally to the neck	1909 Finzi, England [30]	Carcinoma larynx
1910 Combined treatment by radium catheter and external radioactive varnished plaques	1912 Barcat, France [39]	Carcinoma epiglottis
1911	1911 Costiniu, Romania [40]	Carcinoma of the larynx (2 cases)
1911	1912 Price-Brown, USA [41]	Sarcoma of the larynx
1912 insertion of radium tubes	1914 Hill, England [42]	Hypopharyngeal cancer (3 cases)
1909/1913 subhyoid pharyngotomy and direct insertion of radium tube into tumour	1914 Turner, Scotland [43]	Post-cricoid carcinoma involving larynx

Another technical solution for brachytherapy was the use of radon gas for gamma rays which was cheaper and easier to dose than radium. A range of applicators for contact therapy, intracavitary and interstitial use was developed by the biophysicist William Duane (1872–1935) and the first patients were treated in 1913 [44]. Another technical solution were gold radon seeds which were developed by the biophysicist and radiation oncologist Gioaccino Failla (1891–1961) [45]. Finally, interstitial brachytherapy was importantly improved by the surgeon and radiation oncologist Walter Clegg Stevenson (1877–1931) from Dublin in 1914 who loaded a glass capillary with radon gas into a serum needle which can be regarded as a prototype of the later introduced radium needle [46]. The ultimate next steps of interstitial treatment of the larynx however were only introduced in 1923 by the radiologist René Ledoux-Lebard (1879–1948) from Paris who developed the fenestration operation to directly implant radium needles into the tumour and to avoid at the same time the feared necrosis of the cartilage as well as in 1928 by Finzi and Harmer who did not penetrate the airways but packed the radium needles on the intact inner layer of the perichondrium after fenestration [47].

In summary, radiotherapy of larynx cancer was rapidly introduced and technically developed within the first two decades after the discovery of x‑rays and radioactivity in 1895. But organ-preserving radiotherapy of larynx cancer was a therapeutic concept that was introduced on a solid scientific basis only in the 1920 s. Until then, radiotherapy was performed with empirical doses, duration and fractionation chosen individually by the treating physician. Curietherapy dominated the first decades over x‑ray therapy as it was technically less complex to direct the dosis to the tumour and to protect normal tissues.

### Laryngeal cancer in Friedrich III, German emperor

Today, the first steps of radiotherapy of larynx cancer and the fate of the German Emperor Friedrich III (1831–1888) suffering from glottic larynx cancer appear very closely related in time [48]. Obviously Friedrich could not be treated with radiotherapy since he died 7 years before the discovery of x‑rays and radioactivity. Friedrich was the oldest son of emperor Wilhelm I and of Augusta. He was the first Prussian prince with not only a military training but also an academic background, and in 1850 he took up studies of history, politics and law at Bonn University. Among his mentors were the liberal historians Friedrich Christoph Dahlmann and Ernst Moritz Arndt. He spent time in England to meet Victoria the oldest daughter of Queen Victoria, his future spouse who he married in 1858 and who gave birth to Wilhelm in 1859, his oldest son who later became emperor Wilhelm II. During the unification wars of Germany he was a successful leader of the Prussian army but at the same time he despised the cruelties of war. Due to his liberal spirit, his relationships with his father and chancellor Bismarck were difficult, and overall he was politically sidelined. This changed only after his father’s death on 9 March 1888 who he succeeded.

**Fig. 2:**
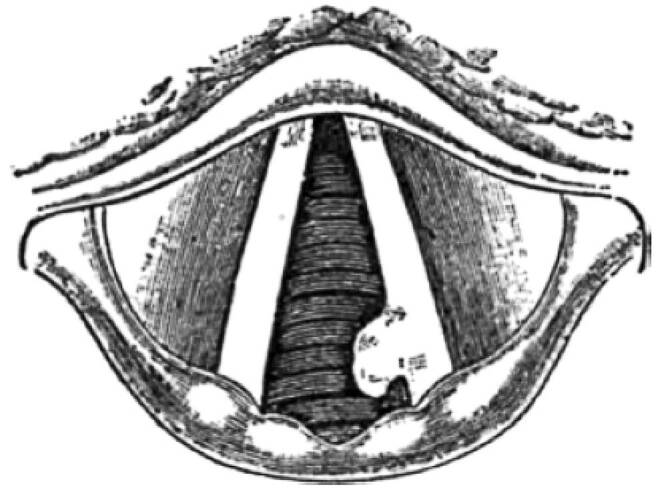
Drawing of the primary examination in May 1887 revealing a left glottic tumour in the posterior third of Emperor Friedrich III by Morell Mackenzie as the cause of his persistent hoarseness. (From: Mackenzie [54])

Friedrich is thought to have taken up smoking during his teens and is thought to have had a 30-year history of tobacco abuse. This is documented by photographs showing him with pipe in hand [49]. However, at that time not smoking but syphilis was commonly accused to be responsible for laryngeal cancer, and there were rumours that Friedrich had contracted this venereal disease during the opening of the Suez canal in Egypt on 17 November 1869 [50]. It appears now to be certain that these rumours have to be regarded as defamation [51]. In autumn 1886, Friederich had a supposed upper respiratory tract infection that resulted in persistent hoarseness (Table 3). Despite of the diagnosis of a 2 × 4 mm nodule on his left vocal cord (T1a) in March 1887 (Fig. 2; [52]), the correct diagnosis of cancer of the larynx was only posed a year later on 4 March 1888, three months before his death from cancer with an autoptic tumour stage pT4 pN1. The details of the course of the disease as summarized in Table 3 reflect that Friedrich could have been cured from laryngeal cancer had the recommendation for the laryngofissure by his primary physicians been followed. In 1887, surgery was the only curative approach for laryngeal cancer. Historians assume that the appointment of Mackenzie from London which was proposed by Dr Wegener and acclaimed by Friedrich’s wife, Victoria, was responsible for the late diagnosis and treatment of Friedrich. An early form of a multidisciplinary tumour board on 6 November 1887 concluded that the course of Friedrich’s hoarseness had to be cancer but by that time the tumour had grown so much that only laryngectomy could have led to a complete resection of the tumour. It is not surprising that Friedrich refused laryngectomy because at that time this surgical procedure still had disastrous effects as shown in an analysis of the first 103 cases [53]. The first successful laryngectomy had only been performed on 24 December 1873 by Theodor Billroth (1829–1894).

**Table 3:** Milestones in laryngology before Friedrich’s illness and timeline of his larynx cancer

*Year*	*Procedure*	*Physician(s)*
1810	Laryngofissure (syn. thyrotomy) developed for foreign bodies extraction	Pierre-Joseph Desault, Paris (surgeon)
1851	Indirect laryngoscopy	Manuel Patricio Rodríguez García, Madrid (opera singer)
1851	Laryngofissure for resection of cancer	Gordon Buck, New York (military plastic surgeon)
1864	Head band light and mirror	Thomas J. Walker, Peterborough (UK; surgeon)
24 December 1873	Laryngectomy	Theodor Billroth, Vienna (surgeon)
1884	Introduction of cocaine anaesthesia	Carl Koller, Vienna (ophthalmologist)
*Year*	*Friedrich’s patient history*	*Physician(s)*
January 1887	Upper respiratory tract infection, persistent hoarseness	August Wegner (medical attendant)
06 March 1887	2 × 4 mm nodule on left true vocal cord	Carl Gerhardt (internist)
29 March–07 April 1887	13 sessions of galvanocautery	Carl Gerhardt (internist)
15 May 1887	Follow-up examination: Recurrent tumour	Carl Gerhardt (internist)
May 1887	Consultation: laryngofissure and resection recommended	Ernst von Bergmann (surgeon)
May 1887	Dr Morell Mackenzie (London) to be consulted	August Wegner (medical attendant) & Victoria (Friedrich’s wife)
20 May 1887	Examination: lesion confirmed but no resection before biopsy and pathology	Morell Mackenzie (laryngologist)
21 May 1887	Laryngofissure (removal and pathology) scheduled but abandoned	2 more panels of consultants
21 May –28 June 1887	4 biopsies (topical anaesthesia with cocaine)	Morell Mackenzie (laryngologist) & Rudolf Virchow (pathologist)
July–October 1887	Conservative therapy: iodine, balneotherapy	Morell Mackenzie (laryngologist)
06 November 1887	‘Tumour board’: has to be malignant, recommendation: laryngectomy—declined by Friedrich	Morell Mackenzie (laryngologist), et al.
09 January 1888	Tracheotomy	Fritz Gustav Bramann (surgeon)
04 March 1888	Histological diagnosis of laryngeal cancer	Wilhelm Waldeyer (anatomist)
09 March 1888	Friedrich III, inaugurated emperor of Germany	–
15 June 1888	Death of Friedrich III from laryngeal cancer	–
16 June 1888	Post-mortem: greater portion of the larynx destroyed by cancer. Today (UICC 8th edition): pT4pN1	Rudolf Virchow (pathologist), Wilhelm Waldeyer (anatomist)

Today, the agreed opinion is that Friedrich III could have been saved from an early death from larynx cancer. A number of problems competed with a determined treatment of his disease. One of them was the failure to establish a clear-cut pathohistological diagnosis. Notably, the involved German and English physicians disagreed on his treatment.

At that time, laryngofissure and resection of the tumour of the left vocal cord at an early stage would have been medically feasible. However, total laryngectomy was incompatible with his function as emperor due to permanent aphonia. Organ-sparing radiotherapy could have reconciled cure with voice preservation had it been available. But obviously, x‑rays were only discovered 7 years after Friedrich’s death, and the first treatment of head and neck cancer with radiotherapy was only performed 8 years after his death. Further development of radiotherapy of larynx cancer shortly after Röntgen’s and Curie’s discoveries reflects scientific progress of medicine. At the same time it demonstrates how the nature of x‑rays and radioactivity had been understood biologically and physically to develop a therapy by stringent development of suitable techniques enabling curative radiotherapy without severe side-effects. As a matter of fact, the path towards this goal was dominated by trial and error since severe side-effects were inevitable in the early days. Consequently, the first attempts to quantify radiation dose consisted in grading acute skin toxicity as a surrogate for tumour dose, and this was termed “skin erythema dose” (German: *Haut-Erythem-Dosis* [HED]) [55].

## Discussion

Arguably, earlier discovery of x‑rays may have prevented the premature death of Friedrich III as well as enthronement of his son and successor Wilhelm II whose political mindset has for a long time been regarded as part of the causes that eventually have paved the way toward World War I. Modern historians such as G. Craig and H. Münkler rather regard him as part of the cogwheel machinery of European politics who was caught in this system.

Unfortunately, the above described hypothetical views of history have to be questioned by justified criticism. While it is tempting to speculate that organ-sparing radiotherapy could have saved the emperor’s voice and cured him at the same time, the true era of curative radiotherapy for larynx cancer started only in 1922, 35 years after Friedrich’s clinical diagnosis of early laryngeal cancer, even if the first patient with head and neck cancer was treated only 8 years after the death of Friedrich III from cancer of the larynx [11, 26]. In addition, the assumption that saving Friedrich in 1888 at age 56 from larynx cancer death would have avoided the start of World War I 26 years later at age 82 is a long shot. Average life expectation of men in 1914 was 45 years and not only health but also political pitfalls are not unlikely to have disempowered Friedrich III by 1914. Furthermore, it is necessary to discuss other oncological modalities of treatment available at the time. These are restricted to surgery and surgical procedures had only just been introduced shortly before Friedrich’s disease. The first surgical cure of malignant disease by surgery is attributed to Jacob da Silva Solis-Cohen by laryngofissure during the American Civil War (1861–1865). This was followed by the first laryngectomy by Billroth in 1873. At the time of Friedrich’s treatment, the mortality rate within the first 8 weeks was about 40% and the recurrence rate was 20% with 8.5% of patients surviving longer than 12 months [56]. This may explain the reluctance for radical surgery at that time in connection with permanent loss of speech.

When Friedrich’s hoarseness persisted after his recovery from a cold in early 1887 laryngoscopy was available to pose a diagnosis after its introduction in 1851 by García in Madrid and refinement in 1864 with head band and mirror by Thomas Walker in Peterborough, England. Thus, on 6 March 1887, Carl Gerhard, a specialist in internal medicine, was able to diagnose a protruding mass from the left true vocal cord. Conservative local treatment at that time consisted in galvanocautery leading to necrosis by heat with an electrosurgical knife, a technique that had been developed by Albrecht Theodor Middeldorpf in Breslau as described by him in 1854. A laryngoscopic control examination on May 15 by Gerhard showed a recurrence which was confirmed by Ernst von Bergemann the next day, and both diagnosed cancer. Consequently, they recommended laryngofissure to resect the small tumour, a technique that had been invented in 1851 by the US surgeon Gordon Buck and successfully used to cure laryngeal cancer by Solis-Cohen as described above. But August Wegener, a medical attendant, and Friedrich’s wife, Victoria, asked for a second opinion since they feared that Gerhard and Bergemann might be wrong with their clinical diagnosis and Friedrich’s voice could be sacrificed without justification. Therefore, Morell McKenzie’s (a London laryngologist) opinion to insist on pathologic confirmation of the clinical diagnosis, by pathohistologic workup of biopsies which he took on May 21 appears very modern from the point of view of 21st century oncologists and is reminiscent to modern multidisciplinary tumour board decisions. As the pathologic diagnosis from these biopsies by the pathologist Rudolf Virchow was pachydermia verrucosa laryngis, a benign disease, aggressive surgery appeared unjustified as suggested by Wegener and by the princess royal. This diagnosis was found to be wrong on March 4, 1888, and corrected to laryngeal cancer by the anatomist Wilhelm Waldeyer which was unfortunately too late to save Friedrich from a by then far advanced tumour. Virchow’s misdiagnosis was later speculated to be hybrid verrucous carcinoma, a rare mixture of pure verrucous carcinoma with clusters of conventional squamous cell carcinoma, which is especially difficult to diagnose [57]. At the time of the false diagnosis in May 1887, radical radiotherapy, could have cured Friedrich from his early cancer which of course was only available from 1896 with a technical and radio-oncological learning curve that reached a certain standard only in 1922 [11, 26]. The more advanced stage of Friedrich’s disease in November 1887 that led McKenzie to revise his diagnosis to be laryngeal cancer would have required laryngectomy as described above. With modern medical techniques, Friedrich could have had a replacement voice by a voice prosthesis, by learning oesophageal speech, or by an electronic speech device. Amazingly, the earliest technique of an artificial larynx was already available at the time when Friedrich was ill.

One of the reasons why it is so attractive to speculate that radiotherapy could have saved not only Friedrich himself but allegedly large parts of the world avoiding World War I is that Friedrich was the incarnation of liberal hope in Prussia and the German Empire. This perception was nourished by the further course of history leading to war under the regency of his son Wilhelm II in contrast to Friedrich. It was thought that Friedrich was influenced a lot by the liberal spirit of his wife, Victoria, and by his studies of history in Bonn. Frederick is known to have been anglophilic not only because of Victoria who was English but also due to his experiences and visits in England. Therefore, it is thought that he would have improved the political relations with England in contrast to his son. This opinion probably was also supported by biographic events of Friedrich: in June 1863 he criticised publicly in Dansk the new restrictions against the freedom of press as non-conformal with the constitution by Bismarck. This led to a severe conflict with his father and also to his withdrawal from politics. In 1866 after the victory against Austria in Königgrätz he objected together with Bismarck against the invasion of Vienna which his father intended. Likewise, he tried to stop the annexation of Alsace and Lorraine after the German–French war in 1871, however in vain. Also, Friedrich welcomed the foundation of the left-liberal “German Free-minded Party” in 1884. Friedrich also called the growing tendency of antisemitism in Germany to be a shame for the country. How high the hopes were that Friedrich would lead Germany towards a more liberal course can also be deduced from the thoughts of Friedrich Nietzsche who saw the early death of Friedrich III as a crucial and decisive tragedy for Germany leading to the loss of final hope of liberal development [58].

After the publication of Friedrich’s diaries in 2012 [59], the myth of a liberal Emperor who would have avoided World War I lost its justification [60]. This is the conclusion of the historian Winfried Baumgart who analysed the diaries. His judgement is that Friedrich was not suitable as an Emperor as he was weak to take decisions and also not strong in his thinking. After 1863, Friedrich was frustrated, became pessimistic and felt useless. He was not only outshone by his father and by chancellor Bismarck but also by his wife Victoria. Friedrich had very outdated ideas of being an Emperor who wanted to reconnect to the Holy Roman Empire of the German Nation. Had he survived his cancer, most probably his wife Victoria would have been Queen and Friedrich the eternal Prince. But also making Wilhelm II the culprit for World War I does not do justice to him as he did not hate England but rather admired and imitated the country of his beloved grandmother.

Nevertheless, this historical analysis might give us an idea of the consequences it may have to save a life from cancer. One famous contemporary example is the actor Michael Douglas who was cured by radical chemoradiotherapy from locally advanced tongue cancer in 2010. After his treatment he acted in 14 movies and won two Golden Globe Awards in 2014 and in 2019. This example can serve us to appreciate the tremendous impact of cancer cure not only for the treated patient himself but also for society.

In conclusion, within a few months after the discovery of x‑rays the first patient with head and neck cancer was treated. Organ-sparing radiotherapy of larynx cancer was developed rapidly thereafter and Friedrich could have been cured by radiotherapy at an early cT1 stage a few years after his tragic death. Radiotherapy rapidly evolved to become the second pillar of oncology after surgery.

## Supplementary Information

Video in MP4 format on otorhinolaryngology (ORL) radiotherapy until World War I, laryngeal cancer of emperor Frederick III and his role in history.
